# The Interplay Between Visceral Adiposity and Cardiometabolic Risk in Children With Obesity

**DOI:** 10.7759/cureus.84062

**Published:** 2025-05-13

**Authors:** Bassma A Abdelhaleem, Ehab K Emam, Ashraf M Salem, George E Yacoub, Rania A Helal, Marwa H Abdelhamed

**Affiliations:** 1 Pediatric Medicine, Faculty of Medicine, Ain Shams University, Cairo, EGY; 2 General Physician, Matarhea Teaching Hospital, Cairo, EGY; 3 Diagnostic and Interventional Radiology, Faculty of Medicine, Ain Shams University, Cairo, EGY

**Keywords:** cardiometabolic risk, obesity, pediatrics, ultrasound, visceral adiposity

## Abstract

Introduction: Lately, pediatric obesity has become a global threat. It is associated with high morbidity and mortality rates from cardiometabolic diseases in young adults. The purpose of the study was to assess the correlation between visceral adipose tissue (VAT) thickness and cardiometabolic risk factors, including insulin resistance, in children and adolescents with obesity.

Methods: A cross-sectional study was conducted on 46 children and adolescents with obesity, with a mean age of 10.26 ± 2.79, from the pediatric clinical nutrition unit of Ain Shams University Hospital, Cairo, Egypt. VAT thickness and subcutaneous adipose tissue (SAT) thickness were measured using ultrasound and correlated with anthropometric measures, fasting lipid profile, homeostasis model assessment for insulin resistance (HOMA-IR), and alanine transaminase (ALT).

Results: VAT thickness measured through ultrasound has demonstrated a positive correlation with body mass index (BMI) and HOMA-IR, with an area under the curve for VAT thickness as a predictor of insulin resistance being 0.858 and 2.98 cm as a cutoff point. In addition, VAT thickness positively correlates with serum triglyceride and ALT levels. Conversely, VAT thickness shows a negative correlation with HDL levels.

Conclusions: VAT thickness measured by ultrasound may be regarded as a suitable prognostic factor for insulin resistance and cardiometabolic risks in children and adolescents with obesity.

## Introduction

The number of children suffering from obesity has increased dramatically in recent years worldwide [1]. This phenomenon is considered one of the biggest health problems among children in this era [2]. In 2022, more than 390 million (20%) of children and adolescents aged five to 19 were overweight, and 160 million (8%) of them were obese. The proportion of overweight and obese children and adolescents aged five to 19 increased dramatically from just 8% in 1990 to 20% in 2022 [3]. The World Obesity Federation also announced that childhood obesity continues to rise, with 254 million children and adolescents aged five to 19 expected to be obese by 2030 [4]. The prevalence of obesity among African school-aged children varies between 4.4% and 21.2% [5]. Pediatric-onset obesity is associated with high morbidity and mortality rates from cardiometabolic diseases in young adults [6].

In the adult age group, the excessive accumulation of visceral adipose tissue (VAT), commonly referred to as visceral obesity, has been linked with metabolic syndrome and is considered an independent risk factor for the development of obesity-related comorbidities [7]. This was explained in some studies by the potential of hypertrophic adipocytes to induce metabolic dysfunction in other cells and tissues of the body, primarily through the aberrant secretion of adipocytokines. It is noteworthy that in children, adipocytokines are predominantly expressed and secreted by VAT rather than subcutaneous adipose tissue (SAT), which serves to underline the important role of VAT in the metabolic health of children [8]. Adipokines, primarily secreted from the adipocytes, may play an important role in obesity-related diseases. They contribute to the balance and control of important body functions and systems such as metabolism, the cardiovascular system, and inflammatory and immunological processes [9]. The dysregulation of adipokines associated with obesity negatively affects many body systems, including energy metabolism and insulin sensitivity, predisposing children with obesity to cardiometabolic disease, nonalcoholic fatty liver disease, and autoimmune diseases [10]. 

Individuals with the same body mass index (BMI) may exhibit disparate VAT thickness and, more worrisome, varying susceptibility to cardiometabolic outcomes. It is imperative to have an accurate, accessible, and reliable tool for measuring visceral adiposity in children and adolescents to facilitate the early identification of cardiometabolic risks [7,11]. Moreover, clinical studies have demonstrated that relying on BMI and other indirect methods for measuring visceral adiposity may be less precise than direct measures, especially in assessing cardiometabolic risks, as they demonstrated a stronger correlation with total body fat distribution than direct measures of visceral adiposity [11].

Several non-invasive radiological techniques have been employed for the purpose of measuring visceral and subcutaneous fat, including computed tomography, dual-energy X-ray absorptiometry, magnetic resonance imaging, and ultrasound. Given its availability, affordability, rapidity, and lack of ionizing radiation, ultrasound is the preferred method over other techniques, particularly in pediatric populations [12,13].

There is a paucity of clinical studies that employed VAT measurements in relation to cardiometabolic risk factors within pediatric age groups. Furthermore, the findings of these studies have been inconclusive regarding the correlation between VAT and dyslipidemia, insulin resistance, and other metabolic risk factors [14].

Thus, the interrelationship between VAT and cardiometabolic risk in the young age group has yet to be the subject of extensive investigation. The primary objective of this study was to assess the correlation between VAT thickness and cardiometabolic risk factors, including insulin resistance, in children with obesity. The secondary objective was to explore the potential use of VAT ultrasound measurements as a prognostic tool for insulin resistance in pediatric populations.

The authors of this article acknowledge Ain Shams University Conference 2023 for publishing the preliminary results as an abstract in their conference proceedings in the special issue (Volume 117) of QJM on April 3, 2024 with the title "Sonographic Evaluation of Visceral Fat and its Correlation with Metabolic and Liver Profiles in Obese Children" [15].

## Materials and methods

Study design and population

This was a single-centered, cross-sectional study. The study was conducted after the approval of the Research Ethics Committee of Ain Shams University, Faculty of Medicine, with approval code MS 447/2021, on August 18, 2021, after the acquisition of informed written consent from the child and/or the caregiver.

The study population consisted of 46 children and adolescents with obesity recruited from the pediatric clinical nutrition unit of Ain Shams University Hospital, Cairo, Egypt, with a mean age of 10.26 ± 2.79 years, ranging between six and 16 years, with 28 (60.9%) females and 18 (39.1%) males. The inclusion criteria were defined as follows: (a) obesity identified by the BMI according to the Centers for Disease Control and Prevention (CDC), where BMI is greater than or equal to the 95th percentile on the growth charts [16], (b) age between six and 16 years (on the evaluation day), and (c) signing a written informed consent form for participation in the study (participants and/or parents/guardians). Exclusion criteria included the presence of any secondary causes (e.g., genetic syndromes, endocrine causes) or complications of obesity (e.g., diabetes mellitus) and the presence of any other chronic diseases.

Study procedures

Clinical Assessment

Medical history was recorded for each participant, including the presence of chronic conditions such as hypertension, diabetes, or any metabolic disorders. Family history of obesity, cardiovascular disease, and type 2 diabetes was also noted. A detailed clinical examination was conducted to assess signs of obesity-related comorbidities.

Anthropometric Assessment

Anthropometric measurements were conducted by two trained personnel in consensus using standardized techniques. All measurements were plotted on the CDC growth charts [16]. Standing height was measured without shoes to the nearest 0.1 cm using a SECA stadiometer. Measurements were done in a righteous position with the feet rotated outside (60o), three points of the back were touching the stadiometer (shoulders, buttocks, and heels), arms hanging at both sides (palms to thighs), and the top flat bar of the device pressed against the vertex of the head [17].

Body weight was measured (to the nearest 0.1 kg) using an electronic scale (Tanita SC-330P, Tanita Corporation, Tokyo, Japan). Being barefoot, the child stood upright at the scale center, unsupported, with the least possible clothing [17]. This was followed by BMI calculation: BMI = weight in kg/height in m^2^ [18].

Waist circumference was measured at the end of expiration (to the nearest millimeter) with a flexible tape being held a horizontal position, parallel to the ground, gently touching the skin without compressing other underlying layers. The tape was placed midway between the lower rib and the iliac crest [19].

Body composition analysis was done by a Tanita SC-330P (Tanita Corporation, Tokyo, Japan) body composition analyzer. Total body fat %, muscle mass %, fat-free mass%, and body water % were measured [20].

Biochemical Assessment

After a 12-hour fast, blood samples were collected by nurses. Samples were obtained into plain sterile tubes. After two hours of standing and centrifugation at 3500 rpm for 10 minutes, blood serum was separated. The serum samples were put in closed plastic laboratory vessels and stored at −18 °C until analysis. Blood samples were collected to measure fasting lipid profile, including total cholesterol (TC), low-density lipoprotein (LDL), high-density lipoprotein (HDL), and triglycerides (TGs) (Au680, Beckman by spectrophotometry technique), serum alanine transaminase (ALT), fasting blood glucose (FBG) (SYNCHRON CX-9 autoanalyzer), fasting insulin (immunometric, chemiluminescent assay on IMMULITE Autoanalyzer; Siemens Medical Solution Diagnostics, Los Angeles, USA), and homeostasis model assessment for insulin resistance (HOMA-IR) score was used as an indicator of insulin resistance = FBG in (mg/dL) x fasting insulin (uIU/mL) /405, HOMA IR up to 1.95 in females and up to 2.06 in males were considered normal [21,22].

Radiological Assessment

**Figure 1:**
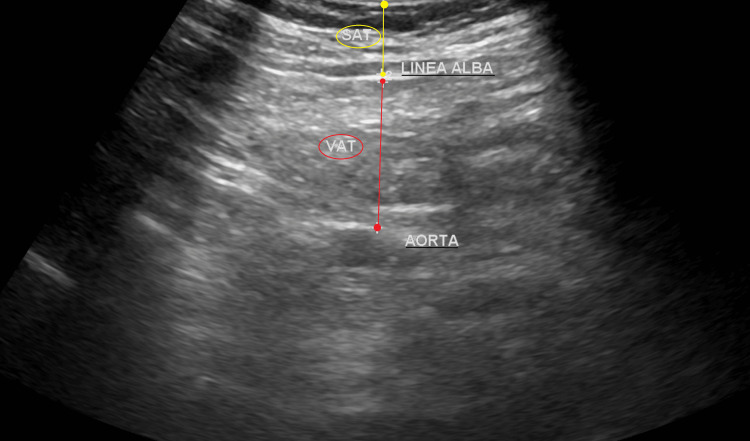
Ultrasound image shows the measurement of the VAT and SAT in a 10-year-old female child. VAT, visceral adipose tissue, SAT, subcutaneous adipose tissue

Measurements were done according to Jung et al. [23] at a 1 cm level above the umbilicus by an experienced pediatric radiologist. VAT thickness was measured using a 3.5 MHz probe from the inner border of the linea alba to the outer border of the abdominal aorta. SAT thickness was measured using a 7.5 MHz probe from the skin-fat interface to the outer border of the linea alba, as shown in Figure 1. Gentle pressure was applied to avoid underestimation of the measurements [23].

Statistical analysis

Statistical analysis was conducted using IBM SPSS Statistics for Windows, version 26.0 (released 2012, IBM Corp., Armonk, NY). The quantitative data were presented as mean ± standard deviation (SD) and range for normally distributed data and as median ± interquartile range (IQR) in addition to range for non-normally distributed data, after using the Kolmogorov-Smirnov test for normality. Qualitative variables were expressed as numbers and percentages. Pearson’s correlation coefficient was used to measure the strength of the linear relationship between variables, where Pearson’s test is suitable to find how two variables are related.

Optimal cutoff points were determined using receiver operating characteristic (ROC) together with sensitivity, specificity, positive predictive value (PPV), and negative predictive value (NPV).

ROC analysis was chosen because it allows for evaluation of the sensitivity and specificity across a range of cutoff points.

The confidence interval was set at 95%, and the margin of error was 5%. All tests were two-tailed, with a significance level set at P ≤ 0.05.

## Results

Descriptive statistics

The study population consisted of 46 children diagnosed with obesity per the CDC definition [16]. Table 1 and Table 2 present an overview of the participant characteristics, anthropometric data, sonographic data, and laboratory data.

**Table 1:** Patient baseline characteristics and anthropometric measures. The data has been presented as mean ± SD or as numbers with percentages in parentheses. n, number of patients, SD, standard deviation

	n = 46	
Age (years)	Mean ± SD	10.26 ± 2.79
	Range	6-16
Gender	Female	28 (60.9%)
	Male	18 (39.1%)
Height (cm)	Mean ± SD	148.20 ± 15.86
	Range	106-174
Weight (kg)	Mean ± SD	68.42 ± 22.39
	Range	24.6-111.7
Body mass index	Mean ± SD	30.15 ± 5.06
Waist circumference (cm)	Mean ± SD	91.08 ± 17.37
	Range	48-119
Total body fat %	Mean ± SD	41.40 ± 6.69
	Range	25.9-58

**Table 2:** Laboratory results and sonographic measurements for the total study population The data have been presented as mean ± SD, median with IQR in parentheses, or numbers with percentages in parentheses. n, number of patients, SD, standard deviation, IQR, Interquartile range, ALT, alanine transaminase, LDL, low-density lipoprotein, HDL, high-density lipoprotein, HOMA-IR, homeostasis model assessment for insulin resistance, IR, insulin resistance, VAT, visceral adipose tissue, SAT, subcutaneous adipose tissue

Measurements		n = 46
ALT (U/l)	Mean ± SD	30.02 ± 14.64
	Range	7-69
Serum cholesterol (mg/dl)	Mean ± SD	160.30 ± 27.54
	Range	70-200
Serum triglycerides (mg/dl)	Mean ± SD	109.41 ± 49.33
	Range	31-238
LDL (mg/dl)	Mean ± SD	93.07 ± 20.66
	Range	29-130
HDL (mg/dl)	Mean ± SD	44.24 ± 9.36
	Range	28-73
Fasting insulin (uIU/ml)	Median (IQR)	13.15 (10.3 - 16.5)
	Range	2.9-37.2
HOMA-IR	Median (IQR)	2.82 (2.19-3.99)
	Range	0.65-9.73
IR	No	10 (21.7%)
	Yes	36 (78.3%)
Fasting blood glucose (mg/dl)	Mean ±SD	94.39 ± 23.48
	Range	70-239
VAT thickness (cm)	Mean ±SD	3.06 ± 0.78
	Range	1.24-5.53
SAT thickness (cm)	Mean ±SD	2.32 ± 0.57
	Range	0.96-3.32

VAT and SAT thickness correlation with other measures of adiposity 

As shown in Table 3, there was a significant positive correlation between VAT thickness and several anthropometric variables, including BMI (P = 0.006), waist circumference (P = 0.002), and total body fat percentage (measured by bioelectrical impedance) (P = 0.001).

**Table 3:** Pearson correlation (r) between VAT thickness, SAT thickness (cm), and other measures of adiposity p < 0.05 is significant (written in bold), Pearson correlation coefficients have been used (r). r, Pearson correlation, VAT, visceral adipose tissue, SAT, subcutaneous adipose tissue

	VAT thickness (cm)		SAT thickness (cm)	
	r	P-value	r	P-value
Age (years)	-0.012	0.937	0.383	0.009
Body mass index	0.397	0.006	0.515	<0.001
Waist circumference (cm)	0.454	0.002	0.322	0.029
Body fat %	0.464	0.001	0.337	0.022

There was a significant positive correlation between SAT thickness and BMI, as well as a positive correlation between SAT thickness and waist circumference (P = 0.002) and total body fat percentage (measured by bioelectrical impedance) (P = 0.001), as shown in Table 3.

VAT and SAT thickness correlation with cardiometabolic risk factors 

Significant positive correlations were observed between the VAT thickness and the HOMA-IR (r = 0.413, P = 0.004), serum TGs (r = 0.421, p = 0.004), fasting insulin (r = 0.305, P = 0.040), LDL level (r = 0.317,p = 0.032), and serum ALT (r = 0.370, P = 0.011). In addition, a significant negative correlation was observed between VAT thickness and HDL (r = -0.313, P = 0.034). There was no significant correlation between VAT thickness and FBG or TC, as shown in Table 4.

**Table 4:** Pearson correlation (r) between VAT thickness, SAT thickness (cm), and cardiometabolic risk factors p < 0.05 is significant (written in bold), Pearson correlation coefficients have been used (r). r, Pearson correlation, VAT, visceral adipose tissue, SAT, subcutaneous adipose tissue, ALT, alanine transaminase, LDL, low-density lipoprotein, HDL, high-density lipoprotein, HOMA-IR, homeostasis model assessment for insulin resistance

	VAT thickness (cm)		SAT thickness (cm)	
	r	P-value	r	P-value
Fasting blood glucose (mg/dl)	0.032	0.831	0.303	0.041
ALT (U/l)	0.370	0.011	0.406	0.005
Serum cholesterol (mg/dl)	0.180	0.230	0.084	0.579
Serum triglycerides (mg/dl)	0.421	0.004	0.517	<0.001
LDL (mg/dl)	0.317	0.032	0.136	0.367
HDL (mg/dl)	-0.313	0.034	-0.006	0.968
Fasting insulin (uIU/mL)	0.305	0.040	0.310	0.036
HOMA-IR	0.413	0.004	0.332	0.024

On the other hand, significant positive correlations were also observed between the SAT thickness and the HOMA-IR (r = 0.332, P = 0.024), serum TGs (r = 0.517, P < 0.001), fasting insulin (r = 0.310, P = 0.036), serum ALT (r = 0.406, p = 0.005), and FBG (r = 0.303, P = 0.041). There was no significant correlation between SAT thickness and HDL or LDL, as shown in Table 4. 

Both VAT thickness and SAT thickness showed no significant correlation with TC, as shown in Table 4.

Cutoff values for insulin resistance

As regards the ROC curve analysis for VAT and SAT thickness as a predictor of IR in all cases, the area under the curve (AUC) of VAT thickness was 0.858 with a cutoff point >2.98 cm. The AUC of SAT thickness was 0.704 with a cutoff point >2.6 cm (Table 5).

**Table 5:** ROC curve of VAT thickness(cm) and SAT thickness (cm) as a predictor of insulin resistance (IR) in all cases ROC, receiver operating characteristic, VAT, visceral adipose tissue, SAT, subcutaneous adipose tissue, AUC, area under the curve, PPV, positive predictive value, NPV, negative predictive value

Parameter	AUC	Cutoff point	Sensitivity	Specificity	PPV	NPV
VAT thickness (cm)	0.858	>2.98	63.89	100.0	100.0	43.5
SAT thickness (cm)	0.704	>2.6	38.89	100.0	100.0	31.2

As for the girls, the AUC of VAT thickness was 0.807, and the cutoff point was >2.69 cm, while for the boys, the AUC of VAT thickness was 0.902, and the cutoff point was >2.98 cm (Table 6).

**Table 6:** ROC curve of VAT thickness (cm) as a predictor of insulin resistance (IR) in females and males ROC, receiver operating characteristic, VAT, visceral adipose tissue, SAT, subcutaneous adipose tissue, AUC, area under the curve, PPV, positive predictive value, NPV, negative predictive value

Parameter	AUC	Cutoff point	Sensitivity	Specificity	PPV	NPV
VAT thickness in females (cm)	0.807	>2.69	77.27	83.33	94.4	50.0
VAT thickness in males (cm)	0.902	>2.98	71.43	100.0	100.0	50.0

## Discussion

BMI is a commonly utilized standard for the assessment of overweight and obesity [24]. However, it should be noted that BMI does not accurately reflect body composition as it cannot differentiate between fat mass and lean mass nor between different types of fat (SAT and VAT), especially in children [11,25]. The distribution of adipose tissue could be considered an independent risk factor for cardiometabolic diseases than total body fat [23]. In the current study, the correlation between VAT thickness using ultrasound and cardiometabolic risk factors in children and adolescents with obesity revealed a positive correlation between VAT thickness and the levels of ALT, LDL, and serum TGs. By contrast, there was a negative correlation between VAT thickness and HDL. SAT thickness exhibited a positive correlation with FBG, ALT, and serum TGs. However, no statistically significant correlation was identified between VAT thickness and FBG, nor between SAT thickness and HDL or LDL.

Our results align with those of Jung et al., who reported a strong positive correlation between VAT thickness and ALT, serum TGs, and a negative correlation with HDL levels [23]. These findings are consistent with those of previous studies that have identified VAT thickness as a significant risk factor for metabolic dysregulation in children and adolescents with obesity [26]. Furthermore, our findings align with those of Dobashi, who discovered a notable correlation between VAT area and TGs, as well as HDL, but not with TC, LDL, or non-HDL-C [27].

Lee et al. [28] reported findings regarding serum TGs and LDL that were comparable to those of the current study. However, the observed positive correlation between VAT and TC, as well as FBG, was not confirmed by the current study. Pak et al. also identified a notable correlation between VAT thickness and serum TGs and LDL, but did not observe a correlation between VAT thickness and HDL, contrasting the findings in the current study [13]. The discrepancy between the study results may be attributed to the differing age groups under investigation, as in the current study the study population ranged between six and 16 years, while Lee et al. [28] the study population ranged between 15 and 17 years and Pak et al. [13] studied younger age group ranged between 2 and 14 years old.

On the other hand, the studies conducted by Burgos et al. [29] and Peçanha et al. [12] did not yield any significant evidence of an association between cardiometabolic biomarkers (TC, FGB, and TGs) and visceral fat. This discrepancy may be attributed to the use of indirect methods (waist circumference and waist-hip ratio) for measuring visceral fat in the study conducted by Burgos et al. [29], which are considered less accurate than the direct methods used in the current study. In addition, the study conducted by Peçanha et al. included only younger, prepubertal age groups [12].

Concerning IR and the HOMA-IR index, our findings revealed a positive correlation between both VAT thickness and SAT thickness with fasting insulin and HOMA-IR. These findings are in accordance with those of prior studies by Lee et al. and Jung et al. [28,23], which similarly demonstrated a positive correlation between VAT thickness and insulin resistance. Also, Peçanha et al observed a significant correlation between the HOMA-IR index and both VAT and SAT, with a notably stronger correlation with SAT [12].

The current study used the ROC curve of VAT thickness and SAT thickness as a predictor of IR in the examined population. The results suggested that the optimal cutoff point for VAT thickness was >2.98 cm for the whole population, >2.98 cm for the males, and >2.69 cm for the females, while the optimal cutoff point for SAT was >2.6 cm for the whole population.

The study's potential limitations include its cross-sectional design, which may not fully capture the nuances of age-related changes. Moreover, the restricted population sample size may limit the generalizability of the findings. Thus, further longitudinal studies with larger sample sizes are still required. In addition, the study encompassed a relatively broad age range, which precluded the ability to reflect the variation in adipose tissue distribution among each age group. Also, the repercussions of puberty on fat distribution were not sufficiently delineated. It is imperative that these issues be addressed in the design of future clinical studies.

## Conclusions

This study presents significant insights into the importance of visceral adiposity assessment in children and adolescents with obesity and its relation to cardiometabolic risks. VAT thickness measured by ultrasound may be regarded as an important marker for cardiometabolic risks, namely, insulin resistance, dyslipidaemia, and elevated liver enzymes, in this specific population.

These findings support the integration of ultrasound in visceral adiposity assessment within clinical practice for the early identification of metabolic risks in this high-risk group, allowing for implying early intervention and prevention policies.
